# Predicting deterioration of patients with early sepsis at the emergency department using continuous heart rate variability analysis: a model-based approach

**DOI:** 10.1186/s13049-023-01078-w

**Published:** 2023-04-01

**Authors:** Raymond J. van Wijk, Vincent M. Quinten, Mathilde C. van Rossum, Hjalmar R. Bouma, Jan C. ter Maaten

**Affiliations:** 1 Emergency Department, University Medical Center Groningen, University of Groningen, Hanzeplein 1, 9713 GZ Groningen, the Netherlands; 2 Biomedical Signals and Systems, University of Twente, Drienerlolaan 5, 7522 NB Enschede, The Netherlands; 3 Cardiovascular and Respiratory Physiology, University of Twente, Drienerlolaan 5, 7522 NB Enschede, The Netherlands; 4 Department of Internal Medicine, University Medical Center Groningen, University of Groningen, Hanzeplein 1, 9713 GZ Groningen, The Netherlands; 5 Department Clinical Pharmacy and Pharmacology, University Medical Center Groningen, University of Groningen, Hanzeplein 1, 9713 GZ Groningen, The Netherlands

**Keywords:** Sepsis, Emergency department, Waveforms, Deterioration, Electrocardiogram, Heart rate variability

## Abstract

**Background:**

Sepsis is a life-threatening disease with an in-hospital mortality rate of approximately 20%. Physicians at the emergency department (ED) have to estimate the risk of deterioration in the coming hours or days and decide whether the patient should be admitted to the general ward, ICU or can be discharged. Current risk stratification tools are based on measurements of vital parameters at a single timepoint. Here, we performed a time, frequency, and trend analysis on continuous electrocardiograms (ECG) at the ED to try and predict deterioration of septic patients.

**Methods:**

Patients were connected to a mobile bedside monitor that continuously recorded ECG waveforms from triage at the ED up to 48 h. Patients were post-hoc stratified into three groups depending on the development of organ dysfunction: no organ dysfunction, stable organ dysfunction or progressive organ dysfunction (i.e., deterioration). Patients with de novo organ dysfunction and those admitted to the ICU or died were also stratified to the group of progressive organ dysfunction. Heart rate variability (HRV) features over time were compared between the three groups.

**Results:**

In total 171 unique ED visits with suspected sepsis were included between January 2017 and December 2018. HRV features were calculated over 5-min time windows and summarized into 3-h intervals for analysis. For each interval, the mean and slope of each feature was calculated. Of all analyzed features, the average of the NN-interval, ultra-low frequency, very low frequency, low frequency and total power were different between the groups at multiple points in time.

**Conclusions:**

We showed that continuous ECG recordings can be automatically analyzed and used to extract HRV features associated with clinical deterioration in sepsis. The predictive accuracy of our current model based on HRV features derived from the ECG only shows the potential of HRV measurements at the ED. Unlike other risk stratification tools employing multiple vital parameters this does not require manual calculation of the score and can be used on continuous data over time.

*Trial registration* The protocol of this study is published by Quinten et al., 2017.

**Supplementary Information:**

The online version contains supplementary material available at 10.1186/s13049-023-01078-w.

## Introduction

Sepsis is a life-threatening disorder with an in-hospital mortality rate of approximately 20%. [1] Depending on the severity of the disorder and the effectivity of the treatment, sepsis can lead to hemodynamic instability, organ dysfunction and eventually death. Early sepsis is a common problem at the emergency department (ED) [2] that can be hard to recognize, but requires timely treatment with antibiotics and fluid resuscitation to prevent deterioration. As a consequence, early detection of sepsis helps providing adequate treatment in time. With the ongoing population aging and related increasing number of comorbidities, the number of patients that present with suspected early sepsis at the emergency department is likely to increase. Physicians working at the ED have to estimate whether the patient is at risk of deterioration and decide whether the patient should be admitted to the general ward, intensive care unit (ICU), or can be discharged home. Over the past decade, several models have been developed to support this clinical decision by risk stratification of patients with sepsis. The Sepsis-2 criteria aims to distinguish the severity of sepsis with the terms sepsis, severe sepsis and septic shock based on the SIRS criteria [3]. With the subsequent introduction of the Sepsis-3 criteria, the quick Sequential Organ Failure Assessment (qSOFA) was introduced as a variant of the SOFA score suitable for the ED. [4] While these models proved to be valuable in patients with severe sepsis and patients at the ICU, their accuracy in early sepsis is inadequate. [5] Currently there is no sufficient tool to adequately recognize early sepsis at the ED.

Both the aforementioned scoring systems rely on measurement of vital parameters at consecutive points in time on specific intervals. Although physicians can compare changes in vital parameters or scores over time, the scoring systems themselves however do not incorporate potentially relevant changes in vital parameters over time. Yet, continuous physiological parameters could provide more detailed information on a patient’s health status. The use of bedside monitors at acute care departments has been common practice for years. With the aid of bedside monitoring devices, physicians have real time insight into vital parameters such as heart rate, peripheral oxygen saturation and blood pressure. Although vital measurements are performed continuously, and repeated measurements are valuable in predicting deterioration [6], the data is often presented as a series of measurements at relatively low frequency. By this way, detail are lost, and long-term changes can be masked [6]; subtle changes or slow trends in vital parameters could easily be overlooked while they could be relevant for identifying development or progression of organ dysfunction. In depth continuous assessment of timeseries data can provide insight in both short- and long-term changes. [7] Heart rate variability (HRV) is a method to interpret the change of a patient heart rate over time and features used in HRV methods can be calculated on a continuous basis. Variability analysis is a way to classify aspects of a complex system, such as the human body. In such system many elements interact with each other, and upon perturbation (e.g. disease) the elements in the system shift. Variability analysis tries to expose these changes by measuring changes to system variables. [8, 9] The concept of beat-to-beat analysis of the heart rate has been around for decades and with ongoing increase of computational power has become more and more relevant. [10] Using both absolute and relative changes of these features, more insight is obtained in the changing health status of a patient instead of relying solely on absolute measurements. HRV is often associated with autonomic balance, blood pressure, respiration and vascular tone. Several features can be derived from beat-to-beat interval and each represent different aspects, for example the sympathetic and parasympathetic balance can be derived from the ratio between low and high-frequency power. Another common interpretation is the reduction of variability, which is associated with the decreased ability to compensate during disease. [11] HRV as a measure to predict deterioration in septic patients has been studied in ICU population and proved valuable in neonatal monitoring. These studies presented models that can predict deterioration in the coming 24 h [12]. A study by Barnaby et al. [13] performed in the ED on a small population, measured HRV over a short period of time and found two frequency components that were associated with severity of illness. Most studies are performed in a relatively sick population with already clear signs of sepsis, while at the ED patients often present in an early stage of sepsis or an uncomplicated infection. It is currently unknown whether HRV can effectively be used to recognize early sepsis in the ED.

We hypothesize that more accurate prediction of deterioration can be achieved, as compared to current risk stratification tools (i.e. SIRS, qSOFA). In this study, up to 48-h of continuously measured electrocardiography (ECG) recordings are used to derive HRV and identify time and frequency HRV features that differentiate deteriorated patients from non-deteriorated patients (i.e. non-progressive disease, recovery). The aim of this study is to identify relevant parameters to associate with clinical deterioration in early septic patients. Those are then used to develop a model to compare to other scoring systems at the ED, such as qSOFA and Sepsis Severity Score.

## Methods

### Population

This observational cohort study was performed in the University Medical Centre Groningen (UMCG, Groningen, the Netherlands), a tertiary care teaching hospital. The study was approved by the institutional review board (METC 2015/164). The study included adult patients that presented at the ED between 8.00 and 23.00 with a suspected infection or sepsis as judged by the attending physician. Patients were only enrolled in case at least two of the SIRS criteria were present: body temperature > 38 °C or < 36 °C, heart rate > 90 beats per minute, respiratory rate > 20 breaths per minute or white blood cell count < 4 10^9^/L or > 12 10^9^/L. Patients were excluded in case of known pregnancy, prior cardiac transplantation or in case they were not admitted to the nursing ward or ICU after triage at the ED.

In accordance with the initial protocol as described here [14], we collected waveforms from bedside monitors and primarily focus on HRV features. The sample size calculation was based on literature and data collected in a previous study at our department [15] and demonstrated a sample size of at least 171 patients. Patients included in the study were connected to a bedside monitor to continuously record waveforms up to 48 h. Progressive organ dysfunction is considered the primary outcome in this study. Patients were post-hoc stratified into three groups, being no organ dysfunction (NOD), stable organ dysfunction (SOD) and progressive organ dysfunction (POD). POD was defined as: development of acute kidney injury (AKI), liver dysfunction or respiratory dysfunction, ICU admission or death within 72 h after ED admission. AKI was defined using KDIGO [16] criteria (i.e., a rise in serum creatinine of 25.6 µmol/L or at least 150%), liver dysfunction was defined as a bilirubin level above 35.2 µmol/L and alkaline phosphatase, ASAT or ALAT more than 2 times normal [17], while respiratory dysfunction was defined based on need of mechanical ventilation or any of the following: PaO_2_ < 8.0 kPa, PaCO2 > 6.5 kPa, SpO_2_ < 90% (ambient air) or SpO2 < 95% (at least 2L/min oxygen supply) [18]. ICU admission was considered based on clinical procedures already in place in this hospital. In general, this was determined by the treating physician at the ED and ICU consultant assessing the patient. The need for vasopressor drugs, invasive ventilation and dialysis in critically ill patients are examples of ICU admission reasons for patients with sepsis in the Netherlands. Patients with kidney, liver or respiratory dysfunction upon admission without development of de novo organ injury (i.e. of another organ as already affected upon ED admission) and without ICU admission or death within 72 h were stratified in the SOD group. Patients who did not show any signs of kidney, liver or respiratory dysfunction at all were put in the NOD group.

### Data collection

ECG waveforms (500 Hz, EASI lead configuration) were captured from ED arrival up to 48 h after arrival using a Philips IntelliVue MP70 with MultiMeasurement module (Philips, Eindhoven, the Netherlands) which was mounted on a semi-mobile cart equipped with a laptop to store the high-resolution waveforms. Three of these mobile monitors were available for this study. Patients were disconnected after 48 h, when they were discharged or declined further measurements.

Data were collected prospectively upon arrival at the ED as well as retrospectively from the electronic patient file to obtain baseline characteristics and allow stratification into the study groups based on outcome. Laboratory measurements were measured at arrival and successively once per day, every morning per current clinical practice. Vital measurements are generally taken multiple times a day, the study followed clinical practice. Organ dysfunction was determined on daily basis using the described criteria. In the case of respiratory dysfunction, when multiple vital measurements per day were available, the worst values were used for that day. Data collected of the first 25 subjects was used in an interim analysis to assess the feasibility of the proposed methods. In this phase practical aspects of data collection were assessed, and algorithms were tested and refined to the proposed methods work as expected. This interim analysis confirmed the proposed methods and did not show any practical limitations and data collection was continued.

### Waveform pre-processing

**Fig. 1:**
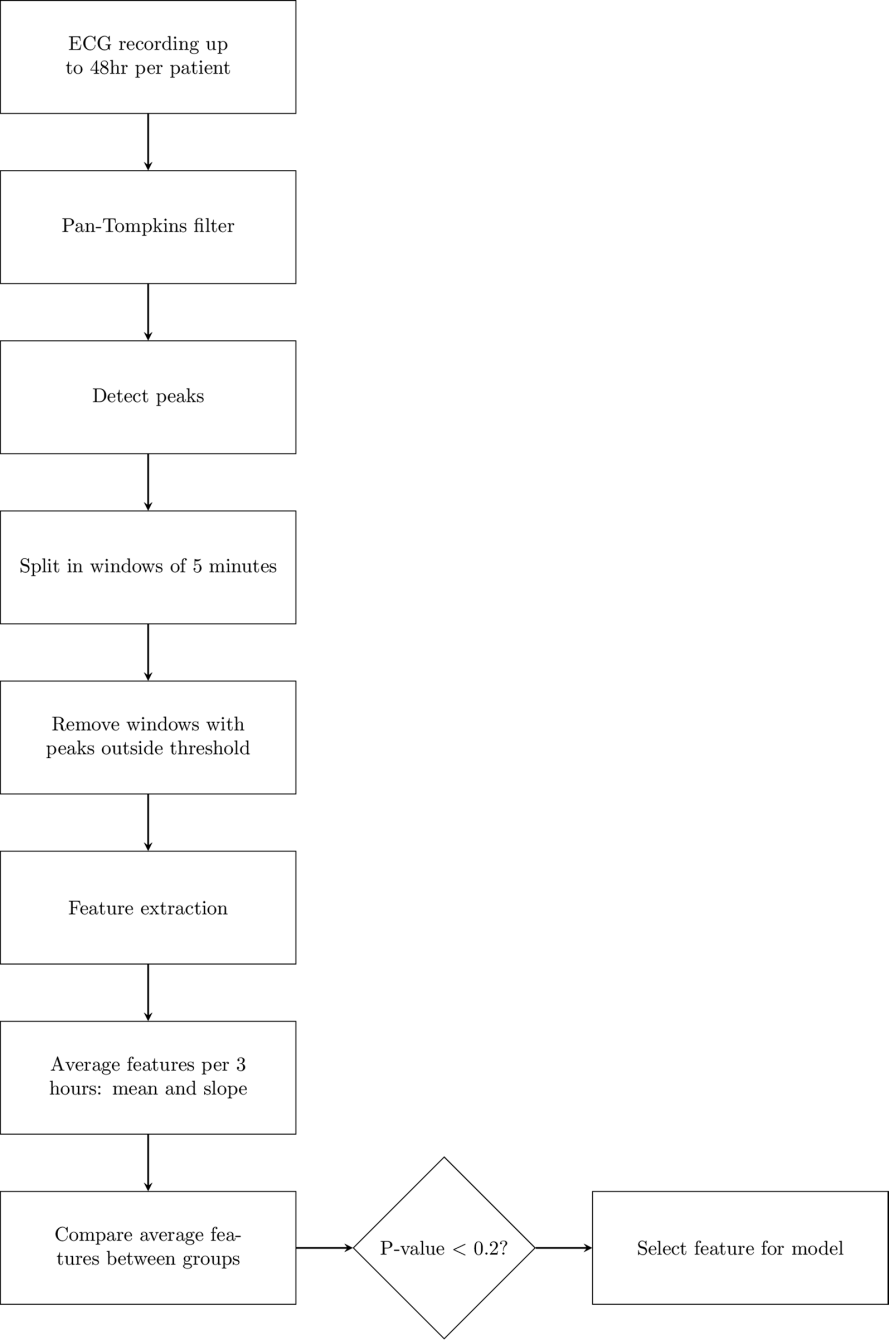
Stepwise overview of the analysis method

Waveforms preprocessing and feature selection steps taken are shown in Fig. 1. HRV is determined by calculating the time between two subsequent heart beats. The resulting tachogram was then used to calculate HRV features in time and frequency domain. A feature is a specific statistical method to calculate the variability over a specific time window. To assess the HRV, continuous ECG recordings were used. All data processing and analysis was performed in Matlab R2018b (Matlab, the Mathworks, Natick, USA). Raw ECG signals from the Philips monitor were first preprocessed using a modified version of the Pan-Tompkins algorithm [19], enabling detection of R-peaks of the ECG and calculation of NN-intervals. Artefacts within the NN-intervals were corrected using the ADARRI method [20]. After preprocessing, the cleaned tachogram was split in subsequent windows of 5 min. Windows with less than 20% of the minimum expected peaks of 300 were removed from further analysis. For each window, a concise set of HRV features as listed in Table 1, were calculated. This leads to 31 features per patient, per 5 min up to 48 h.

**Table 1:** Overview of all features calculated in this study

Name	Description
AVNN	Average of the NN intervals
SDNN	Standard deviation of the NN intervals
RMSSD	Root mean square of successive differences
NN50	Number of NN intervals 50 ms
pNN50	Percentage of NN intervals 50 ms
CV	Coefficient of variation (SDNN divided by AVNN)
SampEn	Sample entropy
SD1	Standard deviation in the first direction of a Poincaré plot
SD2	Standard deviation in the second direction of a Poincaré plot
ULF	Ultra-low frequency power (bandwidth: < 0.003 Hz)
VLF	Very low frequency power (bandwidth: 0.0033–0.04 Hz)
LF	Low frequency power (bandwidth: 0.04–0.15 Hz)
HF	High frequency power (bandwidth: 0.15–0.4 Hz)
Total power	Total frequency power (sum of LF and HF)
LFnorm	LF normalized to total power
HFnorm	HF normalized to total power
LF/HF-ratio	Ratio of LF to HF

The method to extract the features is described in [21]

### Statistical analysis

To analyse the difference in HRV features between the POD, SOD and NOD groups the data was summarized in three hour windows. The windows of all patients were aligned to the start of admission to the ED. This way, patients were all in the same stage of diagnosis and treatment while on the ED. The mean and slope of each feature were calculated per three-hour time windows. A Kruskal–Wallis test was used to calculate differences between the different groups. Significantly different features were then selected for a univariate regression model of each feature to investigate the predictive ability for progressive organ dysfunction. Both the SOD and the NOD groups were considered controls in the model, while progressive organ failure was considered as cases. For this model only the features in the first three hours after admission were used, as they were expected to be useful in prediction at the ED. Next, a stepwise forward multivariate logistic regression model was developed to associate HRV features with the POD group. Features with a p-value < 0.20 in the univariate analysis were used for the multivariate model. For the stepwise regression Akaike’s Information Criterion (AIC) was used to find the optimal model. The resulting model was then compared to models using the qSOFA and Sepsis Severity Score. Statistical analysis was performed and figures were made using Matlab R2018b (Matlab, the Mathworks, Natick, USA) and R for Windows workstations (version 4.0.5) was used for the model, a *p*-value < 0.05 was considered significantly different.

## Results

**Fig. 2:**
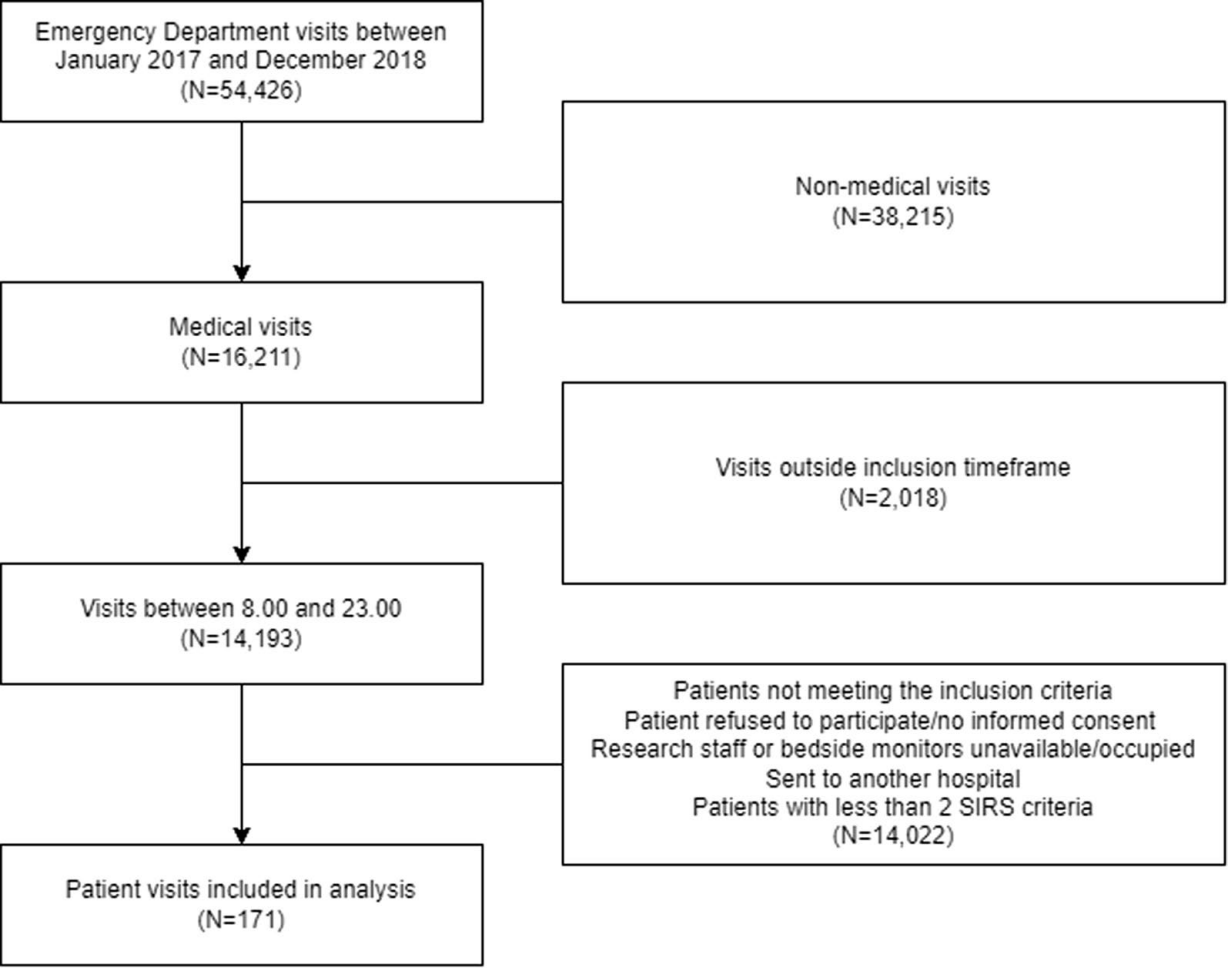
Flowchart of patient visits included in the study

In this study, 169 patients were included between January 2017 and December 2018. Two patients visited the ED two times within the study period, resulting in a total of 171 unique visits. Figure 2 shows a diagram of the included visits. After preprocessing the data, three patients were excluded due to insufficient data availability, which resulted in 168 ECG-recordings for further analysis. Patients were stratified into three groups, being POD, SOD and NOD. Baseline characteristics are shown in Table 2.

**Table 2:** Baseline characteristics

	Stable organ dysfunction (SOD)	Progressive organ dysfunction (POD)	No organ dysfunction (NOD)	*p*
N	38	11	119	
Female (%)	12 (31.6)	4 (36.4)	56 (47.1)	0.22
Age (years)	65 [53, 75]	73 [62, 81]	63 [51, 74]	0.14
Heartrate (bpm)	106 [91, 119]	107 [96, 117]	106 [95, 115]	0.93
Systolic blood pressure (mmHg)	120 [102, 130]	111 [96, 127]	129 [116, 144]	** < 0.01**
Diastolic blood pressure (mmHg)	73 [60, 85]	66 [61, 71]	75 [70, 85]	**0.02**
Mean Arterial Pressure (mmHg)	87 [73, 100]	80 [74, 90]	93 [87, 102]	**0.01**
Respiratory Rate (bpm)	23 [19, 26]	27 [24, 26]	20 [18, 25]	0.14
Peripheral oxygen saturation, SpO2 (%)	96 [94, 98]	96 [91, 98]	96 [95, 98]	0.87
Body temperature (deg C)	38.4 [37.1, 38.9]	38.0 [36.7, 38.5]	38.3 [37.5, 39.2]	0.18
Betablockers (%)	16 (42.1)	1 (9.1)	29 (24.4)	0.05
RAAS blocking (%)	10 (26.3)	3 (27.3)	28 (23.5)	0.87
Diuretics (%)	13 (34.2)	3 (27.3)	27 (22.7)	0.35
Antihypertensives (%)	7 (18.4)	0 (0.0)	15 (12.6)	0.35
Systemic steroids (%)	15 (39.5)	3 (27.3)	38 (31.9)	0.69
Immunosuppressives (%)	12 (31.6)	3 (27.3)	28 (23.5)	0.59
Antibiotics (%)	10 (26.3)	3 (27.3)	30 (25.2)	1.00

Vital parameters were measured upon arrival at the ED. The table shows median and interquartile range (IQR) between brackets for continuous variables and absolute number and percentage for categorical variables. For continuous variables a Kruskal Wallis test was used to test differences between groups and for categorical variables Fisher’s exact test was used. P-values printet bold are below 0.05

**Fig. 3:**
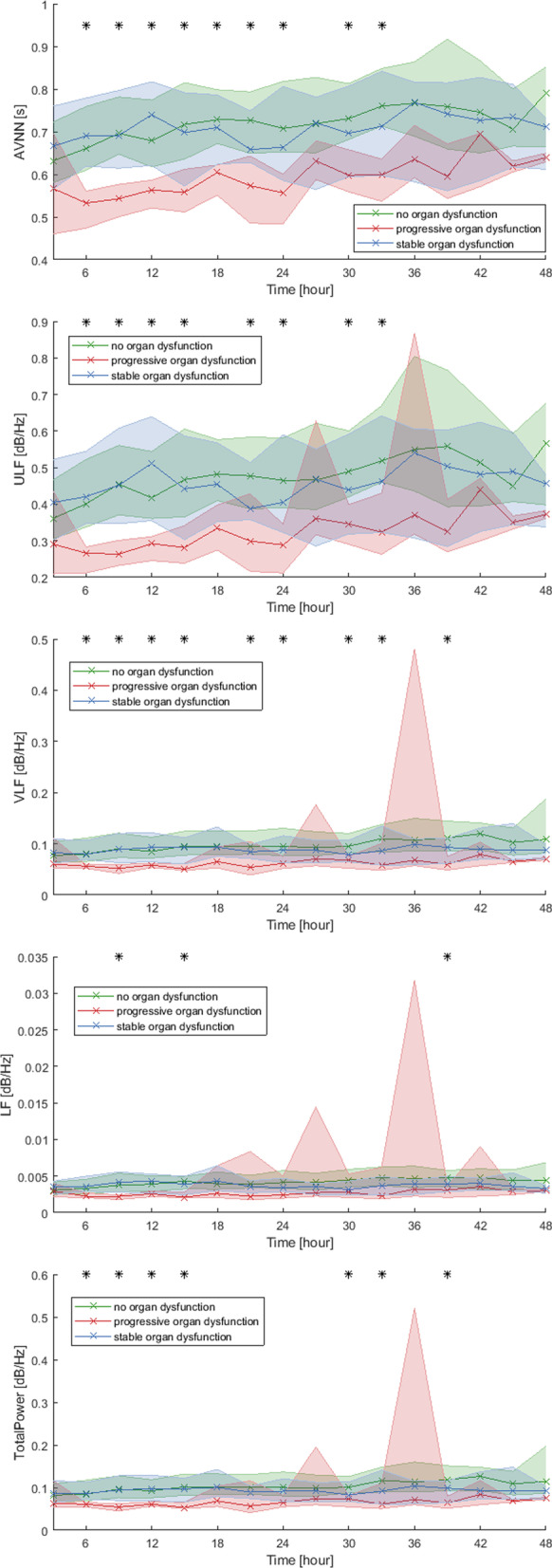
Overview of the means of the HRV features over time. Red indicates the progressive organ dysfunction (POD) group, green the no organ dysfunction (NOD) group and blue the stable organ dysfunction (SOD) group. For each three-hour window the median and interquartile range are shown in the figures as the shaded area. *means *p* < 0.05 between groups

During the first 24 h after ED admission, the three-hour means of the average NN-interval (AVNN) was different between the groups, while ultra and very low frequency (ULF, VLF) and total power were different between the groups during the first 15 h after admission (Fig. 3, Additional file 1: Fig. A1). Low frequency (LF), in contrast, was only different at two three-hour time windows during the first 15 h after ED admission (Fig. 3, Additional file 1: Fig. A1). Most differences between groups were observed during the first 15 h after ED admission, while only a few differences were found between 24 and 48 h after admission. Of note, due to patients being discharged, declining further measurements and mortality, the sample size decreased over time from 168 at t = 0 h, to 105 at t = 12 h, 69 at t = 24 h and 25 at t = 48 h. Comparison of the POD group versus the NOD group showed similar results.

To further analyze whether the change in the HRV features over time can differentiate between patients with POD, from those with SOD and with NOD, we calculated the slope of HRV features and compared the differences between groups (Additional file 2: Fig. A2). The slopes of the features was different a few single points in time, no consecutive points in time where different. Also, none of the slopes was different between admission and 12 h after. In all features, confidence intervals of the SOD and NOD groups are overlapping. The three-hour mean shows features between zero and twelve hours with a distinctive confidence interval in the POD group.

**Fig. 4:**
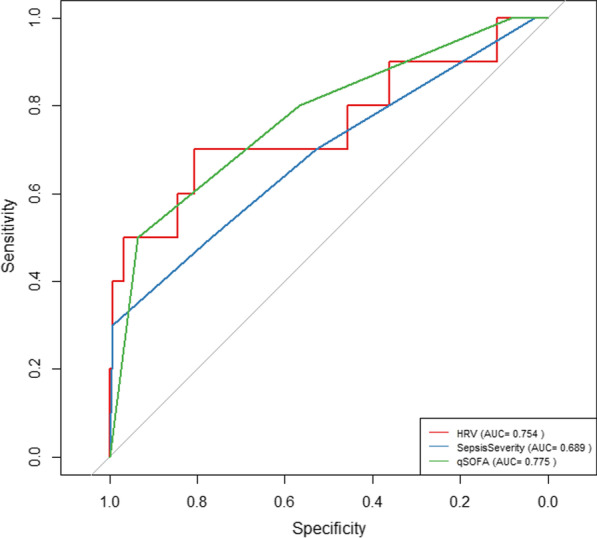
Receiver operating curves of HRV features, qSOFA and sepsis severity score. Receiver operating characteristic of HRV features (red, AUC: 0.754), sepsis severity score (blue, AUC: 0.689) and qSOFA (green, AUC: 0.775). Delong’s test for two correlated ROC curves shows no significant differences between the HRV curve and either SepsisSeverity or qSOFA (p-values: 0.69 and 0.89 respectively)

To compare the predictive accuracy of the HRV features with current risk stratification tools, we did a univariate logistic regression and then developed a multivariate regression model to associate integrated HRV features with progressive organ dysfunction. Selected features and their corresponding relevance to predicting POD are shown in Table 3 and Additional file 3. Next, we constructed receiver-operating curves (ROCs) for the prediction of POD using HRV features, qSOFA and sepsis severity score to compare the area-under-the ROC (AUROC). The AUROC of the multivariate HRV model was 0.754, while the AUROC of the qSOFA and sepsis severity score were 0.775 and 0.689, respectively (Fig. 4). There were no significant differences between the ROC curves of HRV and either Sepsis Severity or qSOFA (p-values: 0.69 and 0.89 respectively) which shows the predictive accuracy was not different between the three scores. Model evaluation showed a R^2^ of 0.195, deviance (-2log-likelihood) of 60.29 with 4 degrees of freedom and a *p*-value of 0.004.

**Table 3:** Univariate logistic regression result

	Univariate			Multivariate		
Characteristic	OR^*1*^	95% CI^1^	*p*-value	OR^1^	95% CI^1^	*p*-value
AVNN	0.00	0.00, 0.61	0.051			
SDNN	5.93	0.00, 34,389	0.7			
NN50	1.00	1.00, 1.01	0.3			
SampEn	0.15	0.01, 1.10	0.090	0.12	0.01, 0.86	0.062
CV	20.2	0.00, 12,825	0.4			
SD2	2.24	0.00, 7,946	0.9			
ULF	0.00	0.00, 1.05	0.085			
VLF	0.01	0.00, 651	0.5			
LF	1.20e + 05	3.473, 1.14e + 14	0.086			
HF	9430	2.699, 1.83e + 10	0.069	2.73e + 26	2.79e + 06, 1.46e + 64	0.048
LFnorm	0.97	0.90, 1.07	0.5			
HFnorm	1.03	0.93, 1.12	0.5			
LFHFratio	0.65	0.34, 1.27	0.2	0.54	0.25, 1.22	0.12
TotalPower	18.4	0.42, 724	0.082	0.00	0.00, 0.02	0.064

Of features calculated between zero and 3 h after admission for progressive organ dysfunction. Variables with *p*-value < 0.20 were selected for multivariate forward stepwise logistic regression, shown on the right

^1^ OR = Odds Ratio, CI = Confidence Interval

## Discussion

To our knowledge, we are the first to identify HRV features that can predict progressive organ dysfunction in early sepsis patients admitted to the ED. Using a mobile bed-side monitor, we collected continuous ECG recordings of 168 unique admissions. POD was defined as de novo kidney, liver or respiratory dysfunction, ICU admission or death within 72 h, and occurred during 11 admissions, while 38 had SOD and 119 did not have organ dysfunction at all. We developed an algorithm to derive HRV features from 48 h long continuous ECG recordings, segmented the continuous measurements in clinically relevant time windows of three hours each and used absolute and trend-based methods to compare HRV features extracted from these ECGs. We demonstrated that AVNN, ULF, VLF, LF and Total Power features differ between the three groups at multiple time-points during the first 12 h after ED admission. We can conclude that each of the scoring methods HRV, SepsisSeverity and qSOFA is predictive of progressive organ failure. Furthermore, the predictive accuracy was not different between the three scores, as demonstrated by comparing the AUROCs of the HRV-score, SepsisSeverity and qSOFA (p > 0.05) Integrating the HRV features into a multivariate prediction model, demonstrated the potential of HRV to be explored as a predictor of progressive organ dysfunction. Yet, as compared to currently used risk stratification tools (e.g. qSOFA), HRV features are extracted only from the ECG and can be relatively continuously obtained from the bedside monitor. For instance, the qSOFA takes measures that have to be obtained by a human, as it contains the Glascow Coma Scale that cannot be measured using a monitor. Using HRV features that can be extracted from the ECG in real-time, measuring intervals can be short (in terms of seconds to minutes) and automated monitoring of the trend is possible. This creates the opportunity to perform trend and variability analysis on a more detailed scale and allows to reveal patterns not seen by human observers investigating multiple individual recordings with a relatively long time interval.

The identification of clinically relevant outcomes for patients with early sepsis at the ED can be challenging, as hard outcomes as ICU admission and in-hospital mortality have a relatively low incidence among patients with early sepsis at the ED, as demonstrated in our population and in line with other studies [6]. Yet, clinical deterioration, defined as progressive organ dysfunction or need to escalate care, occurs more often in this population [6]. Moreover, patients may already have organ dysfunction upon admission to the ED, which can be due to sepsis or chronic co-morbidity, without progression during hospital admission. Therefore, we decided to distinguish between POD and SOD. Since the majority of patients with organ dysfunction had stable organ dysfunction, this resulted in a relatively small group of patients with progressive organ dysfunction. However, by concentrating on this relatively small group with progressive organ dysfunction we were able to identify HRV features that specifically predicted clinical deterioration and differentiate effects on HRV as can be exerted by co-morbidity.

The Sepsis-3 criteria are the current, widely accepted criteria to determine severity of illness in septic patients at the ED and which consist of the qSOFA and SOFA score [22]. However, the tool comprises measurement of vital parameters and laboratory values, usually taken at a single point in time, to estimate disease severity [23]. We revealed that ECG waveforms can be automatically processed to extract HRV features, which can be integrated and used to predict clinical deterioration with similar accuracy as compared to the qSOFA. Integrating HRV features into multi-parameter prediction models could well improve the predictive accuracy above the accuracy of currently available risk stratification tools. By optimizing waveform analysis, for example by exploring alternative window size and interval lengths, trend fitting methods, and combining multiple HRV parameters we expect that HRV analysis has the potential to perform better than current risk stratification tools.

Early prediction of clinical deterioration in patients with sepsis is essential to allow timely initiation of adequate treatment, but supportive data is scarce. The relation between HRV features and clinical outcomes had been investigated before, although other studies either focus on patients presenting at the ED with in a smaller, more severely ill population [13] selected a post-operative population [24] or use general outcome measurements, such as mortality and thereby missed clinical relevant outcomes such as de novo organ failure or ICU admission among survivors [25]. Results from studies among patients with severe sepsis are scientifically very relevant, but of limited clinical relevance for patients with early sepsis at the ED, as severe sepsis is usually reflected by abnormal vital parameters, such as low blood pressure or hypoxemia. Another study among 26 patients with severe sepsis at the ED demonstrated a decrease in LF and increase in HF HRV features as compared to 32 patients with sepsis with these groups defined according to the Sepsis-2 criteria [26]. It shows that both LF and HF are related to sepsis severity. These findings correspond to the decreasing LF/HF-ratio found in the current study. While the time features are mainly based on statistical methods to classify temporal changes, the frequency features are often used to identify sympathetic and parasympathetic balance. The identification of HRV features predictive of clinical deterioration among patients with sepsis at the ED, not limited to those with severe sepsis, allows early identification of patients at risk for deterioration and timely initiation of treatment.

### Strengths and limitations

Several strengths of this study can be considered. First, this study was conducted on a population that is very relevant in clinical practice. By dividing the population in three groups it focusses on distinguishing patients at risk for developing organ dysfunction from patients not at risk. Furthermore, in this study we took continuously measured ECGs to calculate the HRV every five minutes, which enabled us to evaluate the HRV over time and prepares the way for continuous prediction algorithms. A limitation of this study is the fact that HRV is derived from the heart rate only. Characteristics derived from HRV are thus always limited by the information embedded in the heart rate. HRV provides insight in the temporal changes of the heart rate. But the incorporation of other vital parameters such as blood pressure and photoplethysmography are likely to improve models that predict clinical deterioration, due to added dimensionality. Another limitation is the need of monitors used in this study. To obtain continuous, high resolution data for 48 h, we decided to use the standard bed-side monitor (Philips Intellivue) on a cart for this. In the future, once wearable devices become available that are able to capture high-resolution ECG data with sufficient battery life, these data could be obtained using wearable devices. Such devices would not restrict the patients’ freedom of movement and thereby increase patients’ compliance.

During the study three mobile ICU monitors were available to guarantee high quality and high-resolution measurements using a device that is considered standard of care and is able to perform the desired HRV measurements. By default, these bedside monitors are not designed to be mobile. As only three bedside monitors were mounted on a cart for this study, the number of inclusions was limited to the availability of these monitors. We do not expect this to have led to selection bias, as the availability of bed-side monitors and the ability to include a patient did not interfere on patient factors. Although mounted on a mobile cart they did not measure anything when not connected to mains electricity. As result patients who started to recover requested to be disconnected from the monitor to be able to move freely.

### Future perspectives

Early detection of deterioration in patients with sepsis could support clinical decision making in terms of therapeutic decisions and the level of care needed for a specific patient. To reach this goal, further research should focus on better understanding what HRV patterns measured in deteriorating patients mean in relation to deterioration of septic patients as well as development of algorithms that do not rely on hard cut-offs, but rather use patient specific cut-offs. While we employed a one-size-fits all strategy to process and analyze the waveforms, a more patient tailored method could provide a better fit to classify the risk of deterioration. Given that clinical reasoning involved integration of multiple diagnostic information, the results of the HRV data should not be interpreted on its own. We expect that combining HRV data with vital parameters, and potentially also demographic data and laboratory measurements, can increase the performance to predict future deterioration. Furthermore, the use of Artificial Intelligence (AI) in the analysis of HRV parameters may help processing the complex data and finding patterns that are not visible in current analysis techniques. The use of AI in the analysis of continuous waveform data, such as ECG and HRV, can reveal patterns that traditional analysis methods cannot detect. A strong summarization of the recorded data is needed for conventional waveform data analysis, which may result in loss of relevant information. Using AI, new methods to interpret long term measurements are available and therefore the extraction of relevant data can be improved. For instance, algorithms that detect anomalies in continuous measurements could potentially provide valuable information about deterioration. Using such anomaly detection algorithms can improve bed-side detection of a deteriorating patient.

When conducting this study there were no wearable medical devices available that were validated to capture high resolution ECG recordings over 48 h. To be sure that measurements were accurate the standard of care ICU monitors were used (Philips Intellivue). In the future, when more wearable devices are on the market, with a sufficient battery life and these devices show good performance, HRV research could be performed using the same methods and algorithms as well as the mentioned AI techniques. Smart watches are currently unable to record the required ECG data since this currently requires a set of ECG electrodes on the thorax. Wearable devices would increase patients' freedom of movement and thereby increase patients' compliance while performing measurements and participating in a study. This would probably result in longer measurements and fewer dropouts. Furthermore, wearable devices tend to be cheaper than general bed-side monitors, although the latter are generally already available in a hospital. Finally, a larger population with a heterogenous population of early septic patients is needed to perform more robust analysis and to achieve accurate prediction models of clinical deterioration in early septic patients.

## Conclusion

HRV features extracted from the ECG waveform can be automatically processed and predict progressive organ dysfunction, ICU admission and/or mortality among patients with sepsis. Although current analysis only consists of three-hour interval and trend-based analysis, we incorporated a broad range of HRV features to identify predictors for deterioration. The results can therefore be used as a starting point for future studies to select HRV features predictive of deterioration in an early septic population. Integrating the HRV features into multi-parameter models could aid the development of novel, accurate risk stratification tools to predict clinical deterioration among patients with early sepsis and support clinical decision making.

## Supplementary Information

**Additional file 1**. Overview of the means of each feature over time.

**Additional file 2**. Overview of the slope of each feature over time.

**Additional file 3﻿**. First 10-minute model.

## Data Availability

Data is available upon request.
