# Supplementary material for: Predicting deterioration of patients with early sepsis at the emergency department using continuous heart rate variability analysis: a model-based approach
Source: Scand J Trauma Resusc Emerg Med. 2023 Apr 1;31:15. doi: 10.1186/s13049-023-01078-w (PMC10067229; doi:10.1186/s13049-023-01078-w)
Supplement: Supplementary file 2 — Additional file 2. Overview of the slope of each feature over time. [file 13049_2023_1078_MOESM2_ESM.pdf]

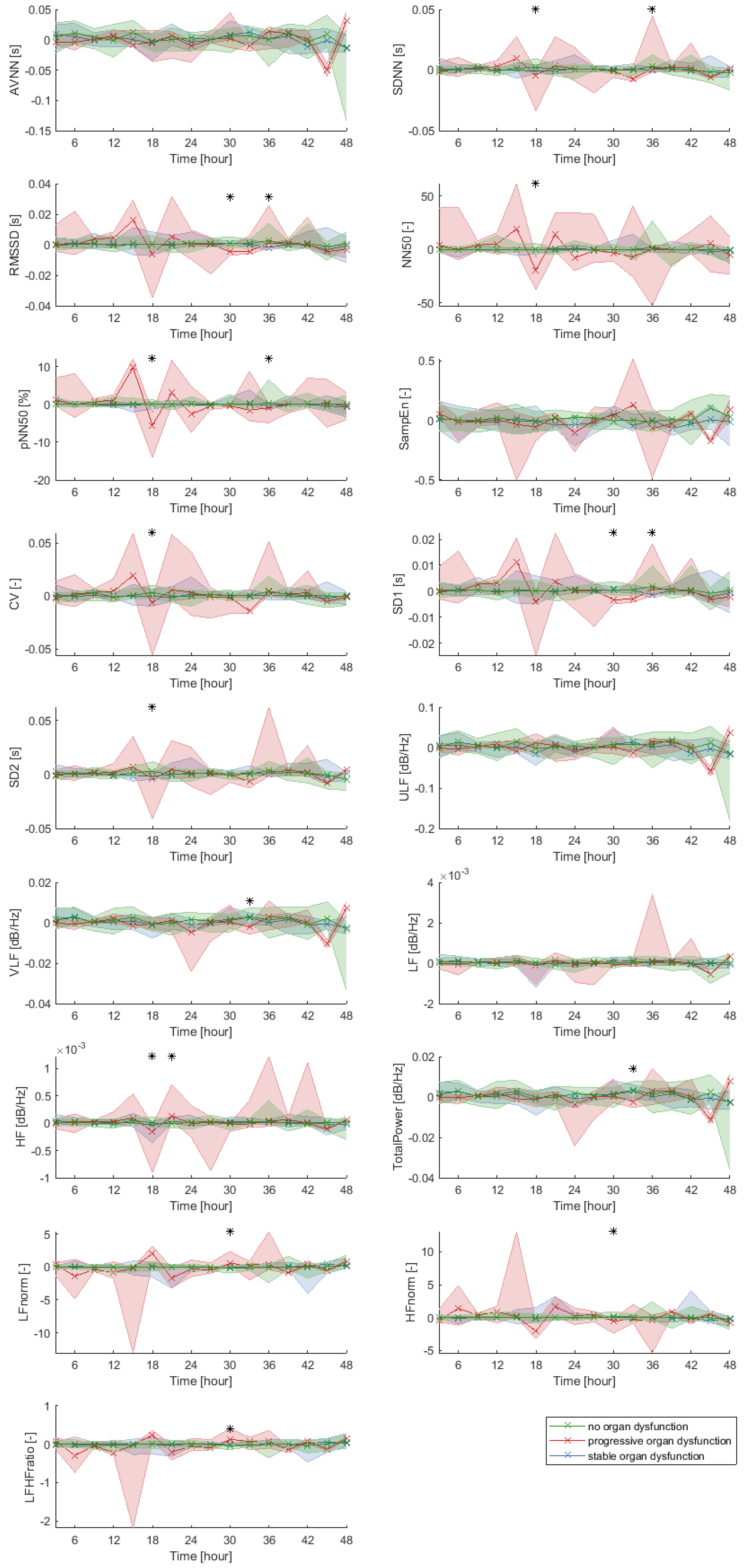

Figure A2: Overview of the slope of each feature over time. Red indicates the progressive organ dysfunction group, green the no organ dysfunction group and blue the stable organ dysfunction group. For each 3-hour window the median and interquartile range are shown in the figures as the shaded area. Measurements that were tested significant ($p<0.05$) using a Kruskal-Wallis test are marked with an asterisk.
