# Supplementary material for: Predicting deterioration of patients with early sepsis at the emergency department using continuous heart rate variability analysis: a model-based approach
Source: Scand J Trauma Resusc Emerg Med. 2023 Apr 1;31:15. doi: 10.1186/s13049-023-01078-w (PMC10067229; doi:10.1186/s13049-023-01078-w)
Supplement: Supplementary file 3 — Additional file 3﻿. First 10-minute model. [file 13049_2023_1078_MOESM3_ESM.pdf]

**First 10-minute model**

A relative short time window of 10 minutes would allow calculation of the features, while on the other hand better matching the workflow at the ED. Hence, we repeated model development using only the first 10 minutes of measurements. Like the original model a univariate regression was done and features with $p < 0.2$ were selected for multivariate regression, as shown in table A1. The AUROC of this model is 0.724, a $R^2$ of 0.093, deviance (-2log-likelihood) of 68.26 with 2 degrees of freedom and a p-value of 0.029.

**Table A1: First 10-minute univariate and multivariate logistic regression result.** Result of a univariate logistic regression of features derived from the first 10 minutes of recording. Variables with p-value < 0.20 were selected for multivariate forward stepwise logistic regression, shown on the right.

| | Univariate | | | Multivariate | | |
|---|---|---|---|---|---|---|
| **Characteristic** | **OR[1]** | **95% CI[1]** | **p-value** | **OR[1]** | **95% CI[1]** | **p-value** |
| AVNN | 0.02 | 0.00, 9.39 | 0.20 | | | |
| SDNN | 8.48 | 1.33, 11,949 | 0.20 | | | |
| NN50 | 1.00 | 1.00, 1.01 | 0.30 | | | |
| SampEn | 0.36 | 0.06, 1.55 | 0.20 | | | |
| CV | 7.09 | 1.31, 2,549 | 0.20 | | | |
| SD2 | 5.34 | 1.24, 1,377 | 0.20 | | | |
| ULF | 0.22 | 0.00, 9.74 | 0.50 | | | |
| VLF | 4.93 | 1.18, 210 | 0.12 | 4.10 | 0.98, 160 | 0.20 |
| LF | 3.18 | 0.98, N/A | 0.80 | | | |
| HF | 2.87 | 0.79, N/A | 0.80 | | | |
| LFnorm | 0.95 | 0.89, 1.03 | 0.20 | | | |
| HFnorm | 1.05 | 0.97, 1.12 | 0.20 | | | |
| LFHFratio | 0.58 | 0.32, 1.05 | 0.06 | 0.61 | 0.34, 1.17 | 0.12 |
| TotalPower | 1.41 | 1.03, N/A | 0.50 | | | |

[1] OR = Odds Ratio, CI = Confidence Interval
